# Hernia fibroblasts lack β-estradiol induced alterations of collagen gene expression

**DOI:** 10.1186/1471-2121-7-36

**Published:** 2006-09-29

**Authors:** Petra Lynen Jansen, Raphael Rosch, Melanie Rezvani, Peter R Mertens, Karsten Junge, Marc Jansen, Uwe Klinge

**Affiliations:** 1 Interdisciplinary Center for Clinical Research Biomat, University Hospital Aachen, Germany; 2 Department of Surgery, University Hospital Aachen, Germany; 3 Department of Nephrology and Clinical Immunology, University Hospital Aachen, Germany

## Abstract

**Background:**

Estrogens are reported to increase type I and type III collagen deposition and to regulate Metalloproteinase 2 (MMP-2) expression. These proteins are reported to be dysregulated in incisional hernia formation resulting in a significantly decreased type I to III ratio. We aimed to evaluate the β-estradiol mediated regulation of type I and type III collagen genes as well as MMP-2 gene expression in fibroblasts derived from patients with or without history of recurrent incisional hernia disease. We compared primary fibroblast cultures from male/female subjects without/without incisional hernia disease.

**Results:**

Incisional hernia fibroblasts (IHFs) revealed a decreased type I/III collagen mRNA ratio. Whereas fibroblasts from healthy female donors responded to β-estradiol, type I and type III gene transcription is not affected in fibroblasts from males or affected females. Furthermore β-estradiol had no influence on the impaired type I to III collagen ratio in fibroblasts from recurrent hernia patients.

**Conclusion:**

Our results suggest that β-estradiol does not restore the imbaired balance of type I/III collagen in incisional hernia fibroblasts. Furthermore, the individual was identified as an independent factor for the β-estradiol induced alterations of collagen gene expression. The observation of gender specific β-estradiol-dependent changes of collagen gene expression in vitro is of significance for future studies of cellular response.

## Background

An impaired collagen metabolism is hypothesized to disturb the wound healing process with the consequence of hernia formation and hernia recurrence. In vivo studies of hernia patients revealed irregularly arranged collagen fibers, disturbed collagen hydroxylation and an altered collagen framework resulting in increased tissue elasticity [1]. Analysis of the extracellular matrix in patients with either primary or recurrent incisional hernias revealed alterations in type I and type III collagen gene expression: a decreased ratio with increased type III collagen protein was detected in incisional hernia patients and was confirmed at the transcript level [2,3]. Alterations of the collagen composition may occur due to changes in collagen synthesis as well as turn-over. For the latter, matrix-degrading proteases may represent key factors. Bellon et al. have suggested that high expression levels of metalloproteinase 2 (MMP-2) play a pathogenetic role for direct inguinal hernia formation [4].

Age above 45 years and male gender are dominant demographic factors that correlate with the incidence of incisional hernia disease and collagen metabolism [5]. Collagen content is significantly decreased in women who have pelvic organ prolapse regardless of age, parity, body mass index, or tobacco use [6]. Furthermore estrogen is a major regulator of wound healing that can reverse age- and sex-related impaired wound healing in human and animal models, characterized by a dampened inflammatory response and increased matrix deposition [7]. Several studies have demonstrated estrogen-mediated effects on collagen and MMP-2 metabolism [8]. Topical estrogen treatment in both males and females decreased wound size with increased collagen levels and enhanced tensile strength [7]. Jorgensen et al. reported increased collagen deposition in premenopausal women after subcutaneous implantation of expanded poly-tetra-fluorethylene test tubes compared to postmenopausal women [9]. Furthermore estrogens influence MMP-2 expression, as MMP-2 levels are reduced after treatment with estrogen in ovariectomized rats [10].

The aim of the present study was to evaluate the immediate effect of β-estradiol on type I and III collagen as well as MMP-2 gene expression in the principal cell type responsible for wound healing that is the fibroblast. An in vitro model system was established with primary fibroblast cultures from patients with and without recurrent incisional hernia disease.

## Results

Collagen type IA1 and IIIA1 expression levels in fibroblasts from patients with recurrent incisional hernia disease compared to control fibroblasts

**Figure 1:**
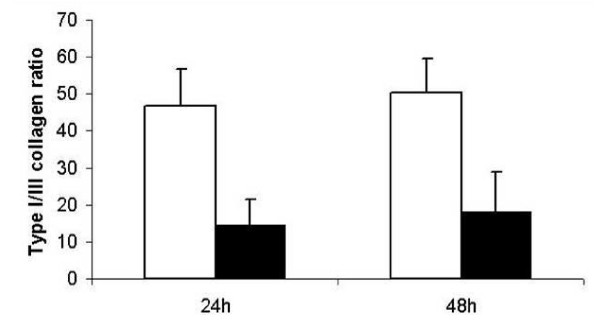
Type I to type III collagen gene expression in primary fibroblasts. Type I/III collagen mRNA (ratio) was quantified by RT-PCR analysis. White bars: CFs (fibroblasts derived from patients without incisional hernia); black bars: IHFs (fibroblasts from patients with recurrent incisional hernias). Data are presented as means +/- standard deviation; * p < 0.05.

The type IA1/IIIA1 collagen ratio was significantly decreased in fibroblasts from male and female patients with incisional hernia disease (14. 5 +/- 7. 0 after 24 hours and 17.9+/- 11. 2 after 48 hours) compared to control fibroblasts from patients without recurrent hernia disease (47. 2 +/- 9. 6 after 24 hours and 50. 6 +/- 9. 2 after 48 hours; Figure 1).

### Changes of collagen expression by β-estradiol stimulation

**Figure 2:**
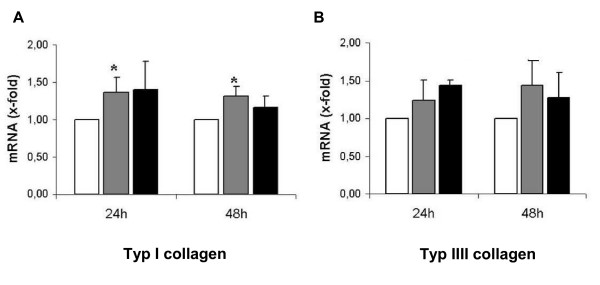
Collagen gene expression in primary fibroblasts from female patient without any history of hernia. Type I and III collagen mRNA was quantified in female CFs (fibroblasts derived from patients without incisional hernia) by RT-PCR analysis. A: Type I collagen mRNA expression; B: Type III collagen mRNA expression; white bars: no estrogen, grey bars: 0.025 ng/ml β-estradiol, black bars: 0.25 ng/ml β-estradiol. Data are presented as mean+/- standard deviation;*p < 0.05 vs. white bars (no estrogen).

**Figure 3:**
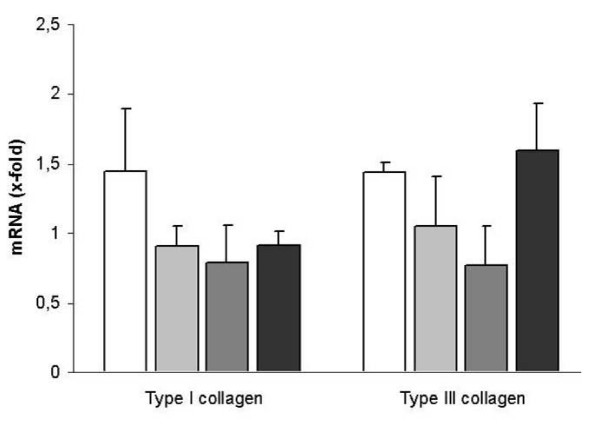
Effect of β-estradiol on type I and type III collagen gene expression. Type I and III collagen mRNA was quantified after 24 h of stimulation with 0.25 ng/ml β-estradiol in female CFs (white bars), male CFs (light grey bars), female IHFs (dark grey bars) and male IHFs (black bars). Data are presented as means +/- standard deviation.

**Figure 4:**
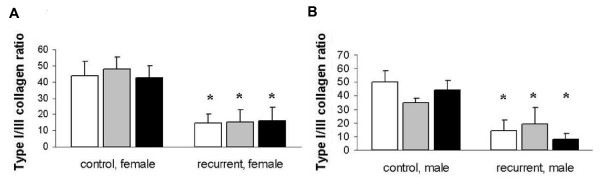
Effect of β-estradiol on type I/III collagen ratio. Type I/III collagen mRNA (ng/μg total RNA) was quantified by RT-PCR analysis after 24 hours of β-estradiol stimulation. White bars: no estrogen, grey bars: 0.025 ng/ml β-estradiol, black bars: 0.25 ng/ml β-estradiol. A: fibroblasts derived from female patients; B: fibroblasts derived from male patients; Data are presented as means +/- standard deviation; *p < 0.05 vers. CFs.

Stimulation with 0.025 ng/ml and 0.25 ng/ml β-estradiol for 24 hours led to increased type IA1 and type IIIA1 collagen mRNA expression levels in fibroblasts from the female control patient (Type IA1 collagen: 1. 36 +/- 0. 21 after 0. 025 ng/ml β-estradiol and 1. 4 +/- 0. 38 after 0.25 ng/ml; Type IIIA1 collagen: 1. 25 +/- 0. 26 after 0.025 ng/ml β-estradiol and 1.44 +/- 0. 07 after 0.25 ng/ml; Figure 2A). This effect lasted for more than 48 hours, however no differences could be detected for the different concentrations of β-estradiol (Type IA1 collagen: 1. 31 +/- 0. 13 after 0. 025 ng/ml β-estradiol and 1. 17 +/- 0. 14 after 0.25 ng/ml; Type IIIA1 collagen: 1. 44 +/- 0. 41 after 0.025 ng/ml β-estradiol and 1.28 +/- 0. 32 after 0.25 ng/ml; Figure 2B). Stimulation with β-estradiol had no effect on type IA1 and type IIIA1 collagen expression in male control fibroblasts (CF) and female incisional hernia fibroblasts (IHF) (Figure 3). Exclusively, male hernia fibroblasts showed high levels of type IIIA1 collagen after stimulation with 0.25 ng/ml β-estradiol for 24 h (1.6 +/- 0. 35 fold increase). The type IA1/III collagen mRNA ratio was significantly lower in fibroblasts derived from patients with incisional hernia disease, both female and male (male IHF vs male CF: 14. 28 +/- 7. 5 vs. 50. 34 +/- 8. 1, p < 0. 05; female IHF vs. female CF: 14. 65 +/- 5. 46 vs. 43. 89 +/- 8. 62), and this ratio was not affected by stimulation with 0.025 and 0.25 ng/ml β-estradiol (Male IHF: 19. 36 +/- 12. 02 and 7. 92 +/- 4. 05; female IHF: 15. 05 +/- 4. 75 and 16. 16 +/- 8. 01; Figure 4). Similarly β-estradiol had no influence on the type IA1/III collagen mRNA ratio in control fibroblasts (Figure 4).

Alterations of MMP-2 expression and enzyme activities in primary fibroblast cultures from patients with recurrent incisional hernia disease compared to control fibroblasts

**Figure 5:**
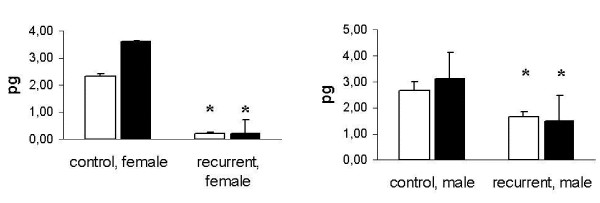
Effect of β-estradiol on MMP-2 gene expression. MMP-2 mRNA (pg/μg total RNA) was quantified by RT-PCR analysis after 24 hours. White bars: fibroblasts without estrogen stimulation; black bars: fibroblasts after 24 hours of stimulation with 0.25 ng/ml β-estradiol. A: fibroblasts derived from female patients; B: fibroblasts derived from male patients; Data are presented as means +/- standard deviation;* p < 0.05 vs. CFs.

The MMP-2 mRNA expression was significantly lower in female and male IHFs compared to CFs (2. 32 pg +/- 0.09 and 2. 65 pg +/- 0.34 in CFs vs 0. 21 pg +/- 0.04 and 1. 66 pg +/- 1 in IHFs; Figure 5A and 5B). Stimulation with 0.25 ng/ml β-estradiol enhanced MMP-2 expression in female as well as male CFs (3. 81 pg +/- 0.04 and 3. 11 pg +/- 0. 18; Figure 5), but not in IHFs (0. 2 pg +/- 0. 5 and 1. 47 +/- 1; Figure 5).

### Estrogen receptor expression

In all primary fibroblast cultures gene expression of estrogen receptor 1 (ESR1, NCB1 gene ID 2099) and, to a minor extend, estrogen receptor 2 (ESR 2, NCB1 gene ID 2100) could be detected.

## Discussion

In the present study the influence of estrogen on the expression of collagen type IA1 and IIIA1 as well as matrix metalloproteinase-2 gene was determined in four different cell cultures. These fibroblasts were obtained from heterogeneous donors that were distinct regarding their history of recurrent hernia disease, sex, BMI and age. The number of cell cultures has to be regarded as a limitation of the study. But besides their heterogeneity, incisional hernia fibroblasts exhibit a reduction of type I to III collagen ratio on the transcriptional level in vivo. These results confirm former studies of our group that described a significantly reduced Type I/III collagen ratio on the protein as well as on the transcriptional level in patients with recurrent incisional hernia disease in vivo [11-14]. It is remarkable that these changes were detected in the absence of any inflammatory cells, pre-existing extracellular matrix and the lack of a three-dimensional cellular network. From this observation it may be concluded that incisional hernia fibroblasts retain an altered phenotype under in vitro conditions and are accessible to ample the influence of steroidal modulation on these cells.

The basic massage of our study is that the individual can be identified as an independent factor for the β-estradiol induced alterations of collagen gene expression. Regarding literature female sex hormones have ample effects on cell growth and the development of connective tissue but in vitro findings are sometimes controversial. Topical estrogen treatment is reported to increase collagen levels, decrease wound size and to enhance tensile strength in both males and female [7]. In accord with this β-estradiol application enhanced collagen gene expression in healthy, female fibroblasts in our study but not in healthy male donors (Figure 3). A sex dependent effect of estrogen was described by Lee et al. who suggested that estrogen effects on collagens is unique to females and might be responsible for the increased incidence of ligaments injury in female athletes [15].

Several other studies suggest that estrogens repress collagen expression: In the rat hip capsule, skin, aorta, and tail tendon long-term estrogen administration causes a decrease in the total amount of collagen [16,17]. Hassager et al. demonstrated that oral estrogen therapy significantly reduces type I procollagen in postmenopausal women and increases total body type III collagen content in a similar group of patients [18,19]. Yu and co-workers established fibroblast cultures from the anterior cruciate ligament and reported a decreased type I procollagen synthesis with increasing estradiol levels whereas no differences in type III collagen synthesis were detected [20]. In accordance we found that β-estradiol at a concentration of 0.025 ng/ml and 0.25 ng/ml had no effect on type IA1/III collagen mRNA ratio in control fibroblasts nor in incisional hernia fibroblasts (Figure 4). In conclusion these date indicate that estrogen is inappropriate to reverse the impaired type I/III collagen ratio in hernia fibroblasts. Regarding these reports, a further decrease of type I/III collagen ratio in dependence of estrogen incubation is absent for hernia fibroblasts and estrogen seems to be of minor importance for the occurrence of the hernia fibroblast phenotype.

The collagens are under control of a degradation system, the main constituents of which are matrix metalloproteinases (MMPs) [21]. MMPs are essential for the proper progression of wound healing [22]. Of note, there is a direct link between MMP-2 and collagen gene expression as collagen binding to discoidin domain receptor II (DDR 2) regulates MMP-2 gene transcription [23]. A former study of our group investigated the biomaterial induced MMP-2 mRNA expression in incisional hernia fibroblasts and detected no alterations of their MMP-2 synthesis compared to control fibroblasts [24]. In contrast, Bellon et al. have suggested that high expression levels of metalloproteinase 2 (MMP-2) in fibroblasts play a pathogenetic role for hernia formation [4]. Here, we found that incisional hernia fibroblasts exhibit a decreased MMP-2 gene expression. One should take into consideration that the epidemiological data of fibroblast donors vary concerning age, BMI and history of smoking or co- medication and this heterogeneity might explain these differences. As hernia disease predominantly occurs in elderly patients it seems difficult to rule out that 'controls' are affected patients in future. Therefore controls should be defined as patients that are older than 60 years and underwent an abdominal incision about a decade ago. Nevertheless, collagen ration proves true as an important diagnostic tool for incisional hernia disease.

Pirila et al. reported, that MMP levels are reduced and type I collagen content is increased in ovariectomized rats after treatment with estrogen [10]. The authors conclude that estrogens promote wound healing due to the inhibition of MMP-mediated collagenolysis. In contrary, Zecchin et al. described that ovariectomy reduces the expression of MMP-2 and type I and III collagen in female rat molar extraction wounds [25]. We found that β-estradiol (0.25 ng/ml) increased MMP-2 in female fibroblasts from healthy donors. Upregulation of MMP-2 expression after estrogen treatment has been described in breast cancer cell and vaginal tissue samples [26,27]. In addition, a decreased MMP-2 gelatinolytic activity has already been described during skin wound healing of OVX rats. The upregulation of MMP-2 and type IA1 and IIIA1 collagen in female fibroblasts confirms the association between estrogen and collagens, and estrogens and MMP-2. Estrogen receptor 1 (ESR1) and estrogen receptor 2 (ESR2), which are present in all our primary fibroblast cultures, might be signal transducers [28].

## Conclusion

Though our in vitro study presents preliminary results, the coherence of MMP-2 inhibition and diminished type IA1 to III collagen ratio in incisional hernia fibroblasts approves that the expression and processing of key molecules in wound healing is disturbed in hernia patients. β-estradiol metabolism seems of minor importance for the impaired collagen metabolism in these patients. In contrast to alterations of collagen metabolism in the range of incisional hernia disease, the individual was identified as an independent factor for the β-estradiol induced alterations of collagen gene expression. The observation of gender specific β-estradiol-dependent changes of collagen gene expression has to be proven in view of the additive effects of age, BMI, smoking or medication for future studies of cellular response.

## Methods

Outgrowths of primary fibroblasts derived from abdominal skin scars of (i) a male patient without any history of hernia; (ii) a female patient without any history of hernia; (iii) a male patient with ten recurrent incisional hernias; and (iv) a female patient with five recurrent incisional hernias were established and analyzed. Patients characteristics are provided in Table 1 (age 62.7 +/- 8.9 years, BMI 24.4 +/- 4.5). All patients were non-smokers. There was no history of steroid medication and no history of familial connective tissue disease. The female patients did not receive any hormone therapy. The study was approved by the local ethics committee and informed consent was obtained from all patients. Scar tissue from control patients were harvested at re-operation excluding acute abdominal cause.

**Table 1:** Demographic data

	Age	BMI	Incisional hernias
Female, control	68	18.6	0
Male, control	72	22.3	0
Female, recurrent	52	28.9	5
Male, recurrent	59	26.4	10
Average	62.7 +/- 8.9	24.05 +/- 4.05	

### Fibroblast cultures and β-estradiol incubation

Immediately after surgical scar excision, specimens were processed for cell culture as previously described [29]. Primary fibroblasts from the 4^th ^to the 10^th ^passage were used. For the investigation of estrogen-dependent alterations of type I collagen, type III collagen and MMP-2 expression, subconfluent (about 60% confluence) fibroblasts were incubated with β-estradiol at 0.25 ng/ml or 0.025 ng/ml (Sigma, Steinheim, Germany) for 24 and 48 hours and harvested thereafter. The chosen concentrations have been reported to represent physiological levels [30]. 48 hours prior to β-estradiol incubation, fibroblasts were held in serum free media (DMEM without phenol red, 0.1% albumin, 0.6% mg/ml glutamine, 100–200 U/ml penicillin, 50–100 μg/ml streptomycin). For collagen assays, cells were cultured in 24-well plates with initial seeding density of 10000 cells/ml. Medium volume: 0.5 ml/1.9 cm^2^. All cell cultures were maintained in a humidified atmosphere at 37°C in 95% air and 5% CO_2_. Experiments were carried out in triplicate with fibroblast cultures of passage 5–10.

### RNA-Isolation and reverse transcription

Total RNA was extracted from fibroblasts (RNeasyMini Kit, Quiagen Operon, Cologne, Germany) and treated with DNase for 20 min (RNase free DNase, Quiagen Operon, Cologne, Germany) in order to avoid amplification by contaminating genomic DNA. Concentration and purity of RNA was determined by measuring absorbance at 260 nm and 280 nm in a biophotometer (Eppendorf, Köln, Germany). Synthesis of cDNA was catalyzed by SuperScript II RNase H-reverse transcriptase-system (Invitrogen, Carlsbad, CA, USA).

### Primers and probes

Oligonucleotide primers and target specific DNA-probes to analyze MMP-2, type IA and type IIIA1 collagen gene expression were chosen using Primer Express Software, Version 1.0 (Applied Biosystems, Forster City, CA, USA) (Table 2) to span exon junctions of the target sequences. TaqMan probes selected were fluorescence-labelled at the 5' end with 6-carboxyfluorescein (FAM) as reporter dye and at the 3' end with 6-carboxytetramethylrhodamine (TAMRA) as quencher. In preliminary experiments, PCR conditions had been optimized regarding concentration of cDNA, primers and target-specific cDNA. Expression of estrogen receptor 1 (ESR1) and estrogen receptor 2 (ESR2) was determined by QuantiTect HS_ESR1 and QuantiTect HS_ESR2 gene expression assays (Qiagen, Cologne, Germany). Cyclophilin was used as endogenous control.

**Table 2:** Primers and probes for TaqMan^R ^analysis

target	primers/probes	sequence (5'-3')
cyclophylline	5-HCYP	GTCTCCTTTGAGCTGTTTGC
	3-HCYP	CCTTATAACCAAATCCTTTCTCTCCA
	HCYP-FAM	TGCTCAGAGCACGAAAATTTTTTGCTGTC
Col1A1	5-COL1A1	ACAGCCGCTTCACCTACAGC
	3-COL1A1	TCAATCACTGTCTTGCCCCA
	COL1A1-FAM	ACTGTCGATGGCTGCACGGAGTCACAC
Col3A1	5-COL3	TCTTGGTCAGCTCTATGCGGA
	3-COL3	TGTCATCGCAGAGAACGGATC
	COL3-FAM	AGAGATGTCTGGAAGCCAGAACCATGCC
MMP 2	5-MMP2	CGCTCAGATCCGTGGTGAG
	3-MMP2	CTTGTCACGTGGGCGTCACA
	MMP2-FAM	CTTCAAGGACCGGTTCATTTGGCG

### TaqMan^R ^reverse transcriptase PCR

Quantification of cyclophilin (hCYP), type IA1 collagen, type IIIA1 collagen, estrogen receptor 1 and 2 (ESR1 and ESR2) and MMP-2 was performed by real time analysis with the ABI Prism 7700 Sequence Detection System (Applied Biosystems, Forster City, CA, USA). PCR reactions were performed in a 96 well microtiter plate with TaqMan universal PCR master mixture (Perkin-Elmer Biosystems) and Platinium Taq-polymerase (Platinium Quantitative PCR SuperMix-UDG, Invitrogen, Carlsbad, CA, USA) under the following conditions: after 2 min at 50°C and 10 min at 95°C samples were submitted to 40 cycles with each cycle consisting of a denaturizing step at 95°C for 15s followed by an annealing step at 60°C for 1 min. Analyses were performed in duplicate. N-uracil-glycosylase restriction was performed to avoid contamination of the sample DNA with other DNA amplificates.

### Quantification of mRNA expression

Type 1A1 and Type IIIA1 Collagen Gene, estrogen receptor 1 and 2 (ESR1 and ESR2):

Levels of mRNA for type IA1 and IIIA1 collagen, ESR1 and ESR2 are expressed semiquantitatively in relative copy numbers normalized against the housekeeping gene cyclophilin (comparative CT method).

### MMP-2 gene expression

Levels of mRNA for MMP-2 were expressed in ng/μg RNA as absolute quantification. To this end a standard curve from serial dilutions of a purified target-specific cDNA was performed for each PCR reaction. cDNA probes were obtained by amplification, cloning into a pGEMT Vector, preparation of plasmid DNA and sequencing.

Data analysis was performed by the Sequence Detection System Software, Version 1.6 (Applied Biosystems, Forster City, CA, USA).

### Statistics

Explorative analysis of data was performed using Statistical Package for Social Sciences (SPSS^®^) – software. The statistical analysis compared three independent experiments and data were organized according to type of primary fibroblast cell culture and β-estradiol stimulation. P values less than 0.05 were considered to be significant. All data are presented as mean values +/- standard deviation.

## Competing interests

The author(s) declare that they have no competing interests.

## Authors' contributions

PLJ carried out the acquisition of data, analysis and interpretation of data, participated in primer and probe design and TaqMan^R ^analyses and drafted the manuscript. RR established the primary cell cultures. MR performed RNA isolation and TaqMan^R ^analyses. PRM participated in the primer and probe design and the transcriptional studies and have given final approval of the version to be published. UK and KJ participated in the design of the study and performed the statistical analysis. MJ conceived of the study, and participated in its design and coordination. All authors read and approved the final manuscript.
